# Supplementary material for: MetaRibo-Seq measures translation in microbiomes
Source: Nat Commun. 2020 Jun 29;11:3268. doi: 10.1038/s41467-020-17081-z (PMC7324362; doi:10.1038/s41467-020-17081-z)
Supplement: Supplementary file 10 — Supplementary Data 7 [file 41467_2020_17081_MOESM10_ESM.zip › File2/Confidence_High_Taxonomy/221937_out.krona.html]

Javascript must be enabled to view this page.

members
magnitude
magnitudeUnassigned
count
unassigned
taxon
rank

221937\_out

55

SRS1041138\_contig\_number\_contig-100\_24074.24075
1

2
superkingdom
54

1239
54
phylum

class
54
186801

186802
order
54

SRS144537\_contig\_number\_44737
1

186803
family
22

1

SRS143342\_contig\_number\_8664
genus
8
572511

species
6
1322

SRS014459\_contig\_number\_40574SRS015065\_contig\_number\_contig-100\_3942.178944SRS019496\_contig\_number\_16575SRS044535\_contig\_number\_contig-100\_7.96616SRS1041137\_contig\_number\_contig-100\_24104.24105SRS104693\_contig\_number\_contig-100\_18.91692


SRS013965\_contig\_number\_7952
species
1
765821

1898203
2
species

SRS017916\_contig\_number\_15103SRS104400\_contig\_number\_38384


SRS013098\_contig\_number\_39382SRS013951\_contig\_number\_39412SRS018541\_contig\_number\_contig-100\_272.94532SRS018623\_contig\_number\_14761SRS019161\_contig\_number\_34030SRS024492\_contig\_number\_contig-100\_1002.43001SRS024549\_contig\_number\_2907SRS024663\_contig\_number\_15182SRS075773\_contig\_number\_29907SRS1041130\_contig\_number\_6061SRS893300\_contig\_number\_6100
species
11
39491

841
1
genus


SRS142503\_contig\_number\_32881
species
1
166486

8
family
186806

1730
8
genus

1263079
species
5

SRS043768\_contig\_number\_28629SRS104975\_contig\_number\_2135SRS142599\_contig\_number\_26157SRS147346\_contig\_number\_60660SRS148511\_contig\_number\_2958


SRS893383\_contig\_number\_31333
142586
1
species

species
2
39496

SRS015663\_contig\_number\_26077SRS051031\_contig\_number\_22882

9
family
31979

1485
genus
9

665940
4
species

SRS045195\_contig\_number\_6398SRS144537\_contig\_number\_42107SRS148874\_contig\_number\_15134SRS149181\_contig\_number\_14571

411486
species
5

SRS014948\_contig\_number\_contig-100\_6861.93421SRS017701\_contig\_number\_20379SRS043841\_contig\_number\_718SRS053649\_contig\_number\_contig-100\_1.145807SRS145497\_contig\_number\_20270

14
family
541000

genus
2
216851

2
species
853

SRS144135\_contig\_number\_15795SRS146812\_contig\_number\_62131

species
2
1898205

SRS078242\_contig\_number\_contig-100\_206.192336SRS149879\_contig\_number\_10839

1952283
9
species

SRS012273\_contig\_number\_46552SRS018049\_contig\_number\_contig-100\_7383.7384SRS019445\_contig\_number\_contig-100\_11195.129877SRS020869\_contig\_number\_contig-100\_40225.75951SRS022071\_contig\_number\_28054SRS045713\_contig\_number\_contig-100\_19270.19270SRS053214\_contig\_number\_contig-100\_29891.29892SRS065504\_contig\_number\_contig-100\_27032.107256SRS144183\_contig\_number\_contig-100\_1597.215474

1263
genus
1


SRS014923\_contig\_number\_7912
2293194
1
species
